# Electrolytic extraction drives volatile fatty acid chain elongation through lactic acid and replaces chemical pH control in thin stillage fermentation

**DOI:** 10.1186/s13068-015-0396-7

**Published:** 2015-12-21

**Authors:** Stephen J. Andersen, Pieter Candry, Thais Basadre, Way Cern Khor, Hugo Roume, Emma Hernandez-Sanabria, Marta Coma, Korneel Rabaey

**Affiliations:** Laboratory of Microbial Ecology and Technology (LabMET), Ghent University, Coupure Links 653, Building A, Room A0.092, B-9000 Ghent, Belgium; Centre for Sustainable Chemical Technologies, University of Bath, Claverton Down, Bath, BA2 7AY UK

**Keywords:** Carboxylate platform, Chain elongation, Biorefinery, Extraction, Electro-fermentation, Membrane electrolysis

## Abstract

**Background:**

Volatile fatty acids (VFA) are building blocks for the chemical industry. Sustainable, biological production is constrained by production and recovery costs, including the need for intensive pH correction. Membrane electrolysis has been developed as an in situ extraction technology tailored to the direct recovery of VFA from fermentation while stabilizing acidogenesis without caustic addition. A current applied across an anion exchange membrane reduces the fermentation broth (catholyte, water reduction: H_2_O + e^−^ → ½ H_2_ + OH^−^) and drives carboxylate ions into a clean, concentrated VFA stream (anolyte, water oxidation: H_2_O → 2e^−^ + 2 H^+^ + O_2_).

**Results:**

In this study, we fermented thin stillage to generate a mixed VFA extract without chemical pH control. Membrane electrolysis (0.1 A, 3.22 ± 0.60 V) extracted 28 ± 6 % of carboxylates generated per day (on a carbon basis) and completely replaced caustic control of pH, with no impact on the total carboxylate production amount or rate. Hydrogen generated from the applied current shifted the fermentation outcome from predominantly C2 and C3 VFA (64 ± 3 % of the total VFA present in the control) to majority of C4 to C6 (70 ± 12 % in the experiment), with identical proportions in the VFA acid extract. A strain related to *Megasphaera elsdenii* (maximum abundance of 57 %), a bacteria capable of producing mid-chain VFA at a high rate, was enriched by the applied current, alongside a stable community of *Lactobacillus* spp. (10 %), enabling chain elongation of VFA through lactic acid. A conversion of 30 ± 5 % VFA produced per sCOD fed (60 ± 10 % of the reactive fraction) was achieved, with a 50 ± 6 % reduction in suspended solids likely by electro-coagulation.

**Conclusions:**

VFA can be extracted directly from a fermentation broth by membrane electrolysis. The electrolytic water reduction products are utilized in the fermentation: OH^−^ is used for pH control without added chemicals, and H_2_ is metabolized by species such as *Megasphaera elsdenii* to produce greater value, more reduced VFA. Electro-fermentation displays promise for generating added value chemical co-products from biorefinery sidestreams and wastes.

**Electronic supplementary material:**

The online version of this article (doi:10.1186/s13068-015-0396-7) contains supplementary material, which is available to authorized users.

## Background

The chemical industry requires a broad range of carbon-based building blocks and platform chemicals, many of which can be generated sustainably through microbial conversions from sugar, lignocellulosic biomass, and carbon dioxide [1–4]. Anaerobic microbial conversions increasingly contribute to the production of sustainable, non-fuel chemicals. In chemistry, greater value is associated with more reactive functional groups [4]. Non-fuel compounds, on average, are priced fifteen times higher per ton than fuels [3], though most anaerobic biotechnology success stories to date are those of bulk bio-fuels such as biogas and alcohols. These chemicals are generally recovered through gas separation or with petrochemical era separation technologies such as distillation and solvent extraction [5]. Though these technologies are mature and well developed for a broad range of chemicals, they are not broadly suited to biologically constrained titers in complex broths, particularly where extensive dewatering is required. The high energy and capital investment often fatally weaken the economics and sustainability of bulk biochemical production. This issue is compounded when production is constrained by a complex substrate, such as agro-industrial sidestreams and waste.

Production and recovery of compounds from a complex substrate of mixed organics require a strategy that includes both the conversion of non-ideal substrates and the targeted separation of products from fractions with overlapping physico-chemical characteristics (e.g. hydrophobicity, volatility) [6, 7]. Volatile fatty acids (VFA) are attractive yet challenging in both substrate conversion and separation, as VFA: (1) are ubiquitously formed as intermediates during the decomposition of organic matter, typically as a mixture of many VFA; (2) are hydrophilic at a more neutral pH and hydrophobic at low pH, allowing solvent extraction; and (3) have a charged carboxyl group under more neutral conditions, allowing electrical motility. Industrially, short-chain linear saturated VFA such as acetic (C2), propionic (C3) and butyric (C4) acid are mostly produced thermochemically. Targeting bio-production of VFA for use as bulk chemicals or chemical precursors (e.g. for conversion to solvents, fuels, polymers) is known as the Carboxylate Platform [8, 9], and is often associated with second generation biorefinery processes and sustainable substrates such as syngas, and agro-industrial residues and sidestreams. Separation and recovery technology is recognized as a major challenge within the Carboxylate Platform due to the aforementioned unit operation technological hurdles, but also due to product inhibition [10, 11]. Short to mid-chain VFA (C6–C8) acids are toxic at relatively low concentrations, but are commonly targeted for their high added value. Chain elongation to caproic acid (C6) has been demonstrated with mixed microbial communities on both synthetic feed and real streams, with *Clostridium kluyveri* and *Ruminococci* often identified as key players in the microbial community [12–17]. *Megasphaera elsdenii* has been shown to generate caproic acid from sugars and lactate as a pure culture [18–21]. Active removal of caproic and heptanoic acid is critical in sustaining production [15, 17, 20].

**Fig. 1:**
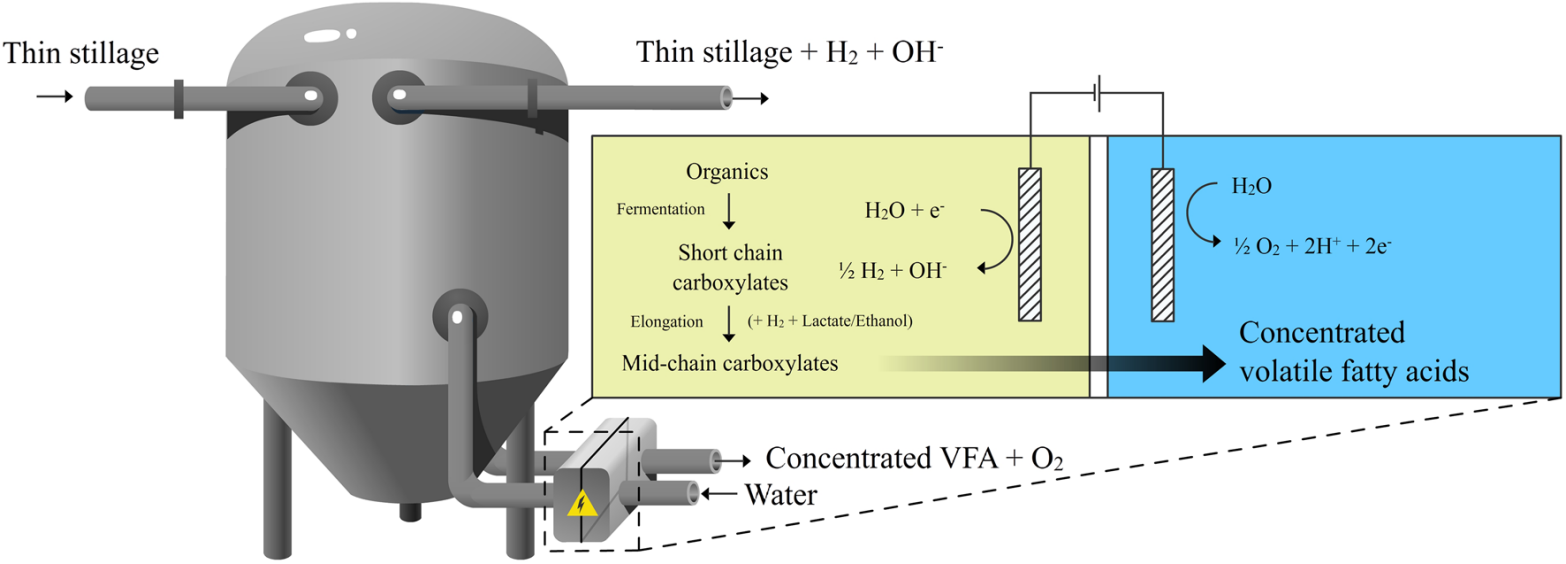
Schematic of electro-fermentation and membrane electrolysis

Membrane electrolysis is an electrochemical extraction technique demonstrated for carboxylate recovery and concentration of short-chain VFA [22, 23] and phase separation of caproic acid [17]. In short, charged products flux across an ion exchange membrane, driven by the electrolysis of water in both the fermentation and the extraction compartment (Fig. 1). Hydrogen gas is produced at the cathode in membrane electrolysis, creating a surplus of biological reducing equivalents that may drive reverse β-oxidation and VFA chain elongation [24, 25]. Other than hydrogen (H_2_), the electrolysis products of protons (H^+^) and hydroxide ions (OH^-^) can be utilized as acid and base without the addition of salts. Hydroxide can counter acidogenic fermentation, while protons generated at the anode acidify the extracted carboxylate, allowing acid accumulation [17, 22, 23]. Andersen et al. demonstrated acetate extraction in a synthetic broth with a high extraction efficiency for a concentration of around 10 g L^−1^, with efficiency decreasing at lower concentrations [22]. Gildemyn et al. demonstrated combined acetate production (microbial electrosynthesis) and extraction with membrane electrolysis, using homoacetogens enriched for hydrogen metabolism and low applied current [23]. Xu et al. used membrane electrolysis to extract, acidify and phase separate caproic acid from a chain elongation reactor; however, the membrane electrolysis was separated from the broth by two units (solids separation, liquid–liquid membrane extraction) and did not interact directly with the fermentation [17].

The unconverted organic fraction of bioethanol production from food crops is a rich, untapped source of complex organic compounds [26, 27], with as much as half or more of the carbon entering the system remaining unconverted [3]. This unconverted fraction is most commonly directed toward distillers grains (also known as ‘dried distillers grains with solubles’, DDGS), a low value agricultural feed product that is an integral co-product in modern bioethanol refineries [28, 29]. In this study, we target thin stillage, the liquid fraction separated from the whole stillage (the distillation column bottoms in a bioethanol production). Though it varies depending on the size and operation of the plant, thin stillage is generated in the vicinity of 10^5^ tons per year per plant and retains a high portion of solids, between 0.1 and 1 %, that are directed back to the production of DDGS after dewatering. Targeting the fermentation of thin stillage allows for a bio-production strategy on an organic rich, low impact stream, and avoids the embedded energy cost (e.g. distillation) in performing VFA chain elongation with recovered VFA and ethanol or lactic substrates.

Petrochemical era extractions typically sit downstream of the production process, while membrane electrolysis interacts directly with the fermentation for maximum utility of the input energy. It is therefore critical that the process implications of membrane electrolysis on the fermentation are understood in detail. Our study explored the impact of in situ membrane electrolysis on VFA fermentation of a real, untreated biorefinery stream. We characterized the impact of the electrolysis products (hydrogen, hydroxide) on thin stillage and the fermentative bacterial community native to this stream. We relate this to changes in the fermentation process, and discuss how this may impact sustainable VFA production.

## Results and discussion

### Stable fermentation with extraction and electrochemical pH control

**Fig. 2:**
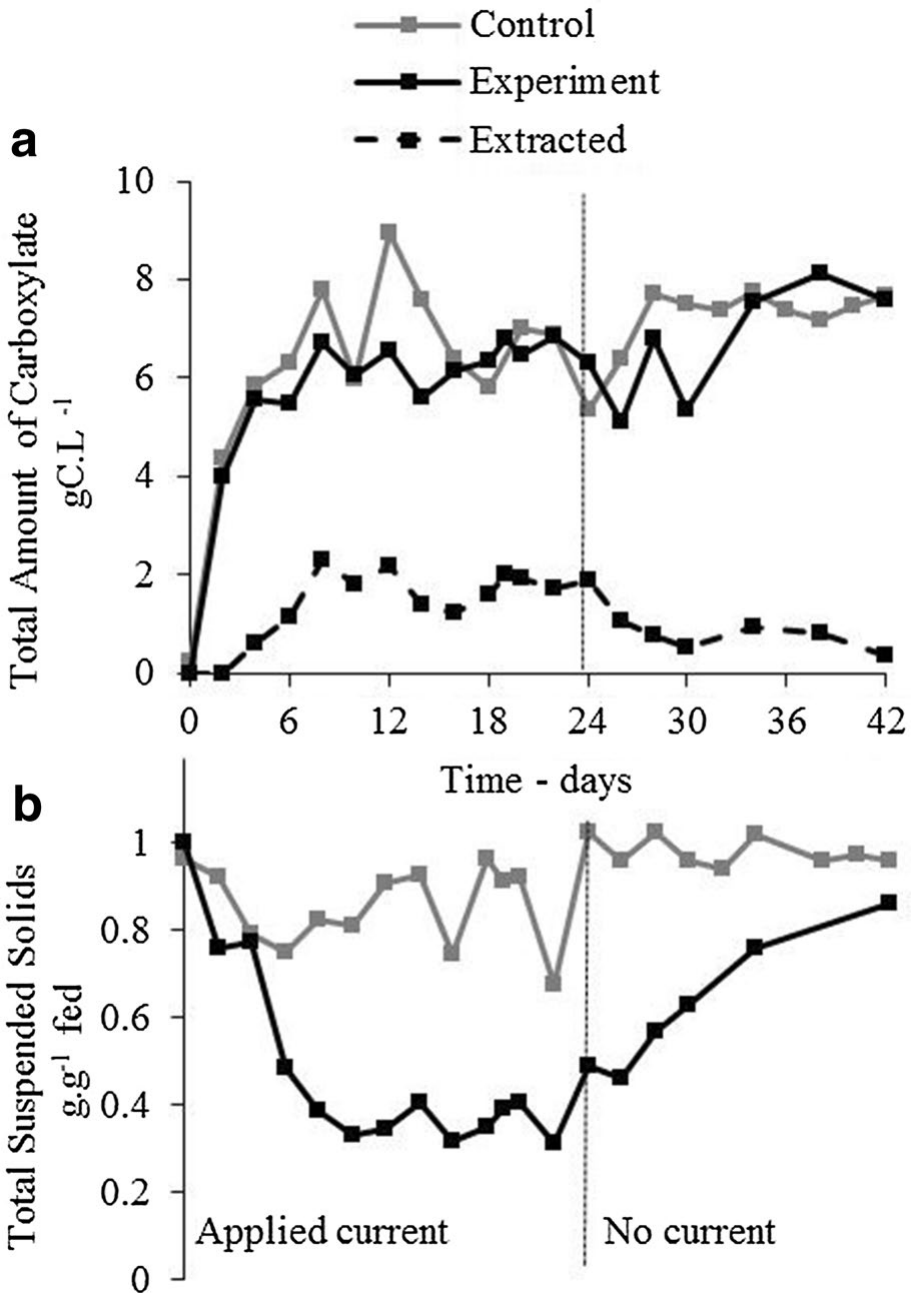
Control and experimental fermentation over time; total amount of carboxylates and total suspended solids. In the experimental fermentation, current was applied prior to the vertical dotted line. **a** The total amount of measured carboxylates. Note the experimental case includes the amount extracted. **b** Total suspended solids measured represented by the proportion of total suspended solids in the fermenter relative to that of the feed

**Fig. 3:**
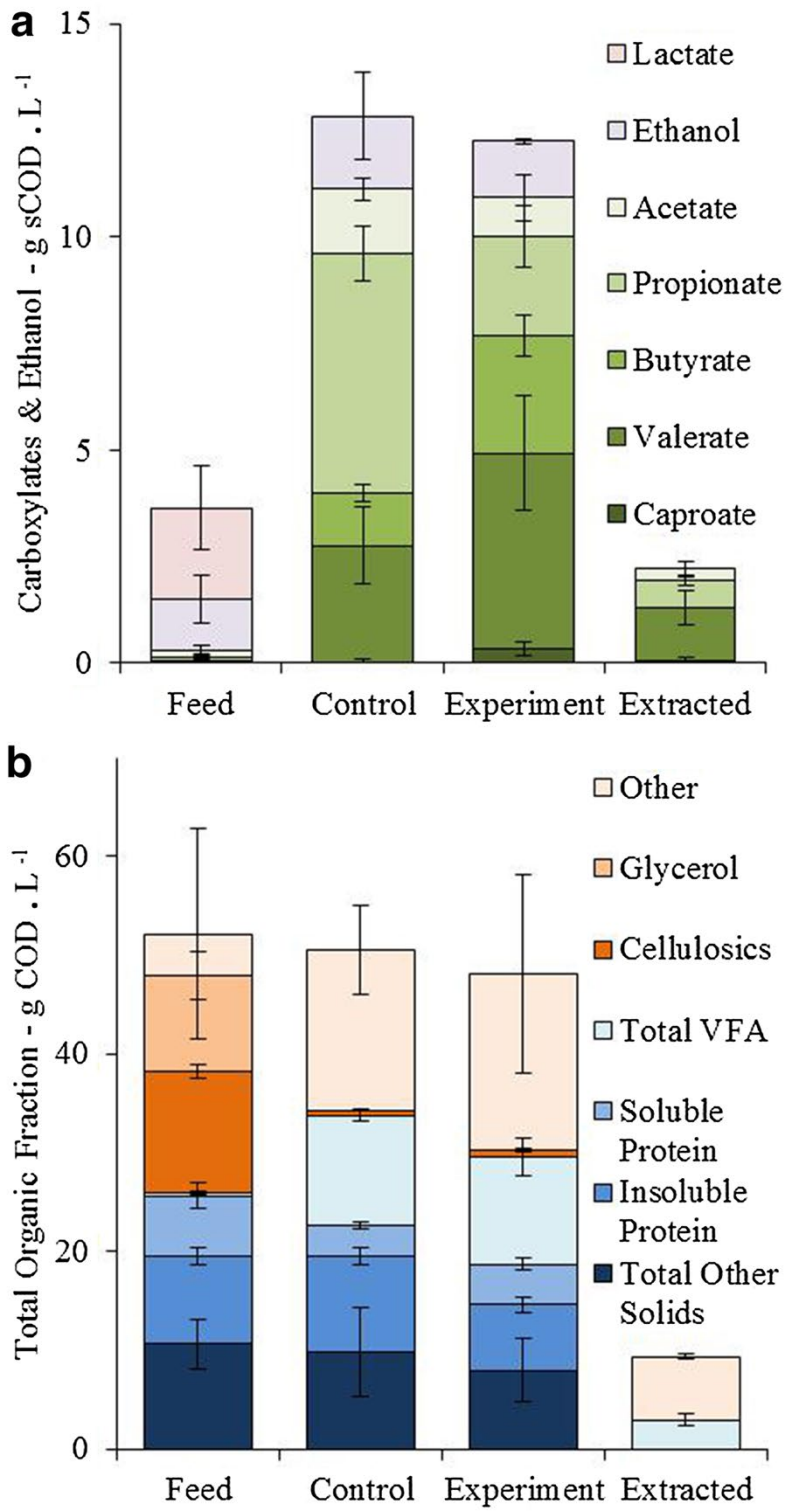
Carbon oxygen demand (COD) balance in the feed, control fermentation, experiment fermentation (applied current) and in the extractant, measured during steady state (day 6–24). Note the extractant is also considered in the experiment column. **a** Total amount of measured carboxylates and ethanol. **b** Total amount of all identified components (*n* = 9 for carboxylates, *n* = 4 for other components, per stream)

Thin stillage was semi-continuously fermented under control and experimental (applied current) conditions, with membrane electrolysis extraction in the experimental case. No difference was observed between the total VFA produced at steady state under control conditions (Fig. 2a). The control is defined as the identical fermentation without an applied current, with sodium hydroxide supplied for pH control, while the experimental case had an applied current (100 mA, 3.22 ± 0.6 V, approximately 20–24 h) until the upper pH set point of pH 5.7 was reached, and then the current was lowered until the next feeding (20 mA, 2.36 ± 0.25 V, approximately 24–28 h) to prevent pH overshoot. Where the pH exceeded the upper set point, VFA rich acid from the anolyte was dosed back into the reactor, though this occurred on average at less than 1 mL day^−1^. The average production rate of short-chain linear unsaturated C2–C7 VFA in the control and the experimental case were similar at 1.9 ± 0.8 g COD L^−1^ day^−1^ (2.5 ± 1.0 gC L^−1^ day^−1^) and 1.9 ± 0.5 g COD L^−1^ day^−1^ (2.3 ± 0.6 gC L^−1^ day^−1^), respectively, at the 6-day hydraulic retention time (HRT) condition. In both the control and experimental fermentation, a similar maximum amount of VFA was generated per total initial volume of thin stillage, with a total conversion of 31 ± 2 % sCOD for the control and 30 ± 5 % sCOD for the experiment (Fig. 3). If the sCOD that can be attributed to protein and oils is excluded, the conversion was 44 ± 2 % for the control and 43 ± 7 % for the applied current fermentation on a sCOD basis. The broth appears to have approached the maximum conversion to VFA under these reactor conditions. Membrane electrolysis extracted 28 ± 6 % of carboxylates generated (rate of extraction/rate of production). The extraction rate was not optimized in this study, which pertains more to electrochemical reactor design and operation (e.g. solids separation, membrane crossflow velocity, surface convection, optimized applied current scheme). The membrane flux rate is closely linked with the total molar concentration in the broth, as demonstrated in previous work involving extraction efficiency [22]. In this study, we focused on the effect of the electrolysis products on the fermentation rather than the effect of the extraction.

The applied current for membrane electrolysis will cause a potential loss due to membrane fouling and solids between the electrode and the membrane. Current interrupt experiments investigated the change in resistance and the ohmic drop over a 5-day period due to solids buildup between the electrode and the membrane. The resistance attributed to the region between the electrode and the membrane was calculated at 3 Ω from the current interrupt experiments at the beginning of the experiment (new membrane), and increased to 10 Ω after 1 day. The resistance peaked at 19 Ω after 4 days. Cleaning the space between the membrane and the electrode decreased this resistance to 10 Ω. Solids buildup on the electrode and membrane can therefore account for almost 1 V of ohmic drop, representing a third of the applied potential under experimental conditions of 100 mA at 3.22 ± 0.6 V. An improved flow design or cleaning regime could translate into a power savings on membrane electrolysis, with increased cross flow velocity likely to improve anion flux.

Acidification of the fermentation broth was completely avoided by the cathodic generation of hydroxide for a zero chemical input pH control. Upper limit pH control was managed by dosing acid extractant back into the fermentation broth, at less than 1 mL day^−1^ on average, as the applied current scheme was designed to avoid excess electrolysis. In the control case, 24 ± 17 mL day^−1^ of 2 M NaOH was added to maintain the pH, equivalent to approximately 10 kg day^−1^ of caustic soda per cubic meter of thin stillage fed. For each kilogram of COD_VFA_ generated this equates to 0.83 kg of sodium hydroxide required to manage the fermentation. At the anode, the oxidation of water generates oxygen gas and protons in the anolyte (extractant), resulting in the protonation of the carboxylate and other anions (phosphate, chloride, sulfate, etc.) that have crossed the AEM.

The experimental reactor decreases in total suspended solids (TSS) relative to the control case after current is applied (Fig. 2b), stabilizing at 0.36 ± 0.04 g.g^−1^ TSS fed, relative to the control case of 0.86 ± 0.11 g.g^−1^ TSS fed in the control case during the steady state (solids content is reported here as grams per liter of TSS_Reactor_/gram per liter of TSS_Feed_ fed due to inconsistency of solids in the fed thin stillage, i.e. minimum 14 g TSS L^−1^, maximum 25 g TSS L^−1^.) The total suspended solids in the control stabilized at a rate of 0.7 ± 1.4 g TSS L^−1^ day^−1^ compared to the applied current case which decreased at an average rate of 3.9 ± 1.9 g TSS L^−1^ day^−1^ in the steady state period from day 8 to day 24. This corresponds to a 4.5 times greater decrease in suspended solids, albeit with large variability. The current was stopped at day 24 to confirm the effect. The stoppage coincided with the suspended solids concentration returning to that of the feed. No equivalent decrease in the measured solids COD nor increase in measured soluble cellulosic fragments or VFA production was observed. This phenomenon is likely related to electro-coagulation, in which the applied current is neutralizing suspended particles, resulting in the formation of coagulated particles [30]. Some evidence to support this is revealed in nitrogen analysis, where 85 ± 6 % of total fed nitrogen remains in the broth in the control case, whereas 71 ± 5 % of total fed nitrogen is measured in the broth in the experimental case, with this difference accounted for mainly in the insoluble nitrogen compounds. Protein or lignin polymers can form colloids at high pH, which may adsorb at the high local pH on the cathode surface, or settle within the electrochemical cell. More research is required into the electro-coagulation phenomenon and the implication for the bacteria and their access to the substrate. If membrane electrolysis is applied in a similar broth in which conversion of solids are targeted, it is not clear if the apparent electro-coagulation will have an effect on the ability of the microbial community to metabolize these solids.

### Fermentation and chain elongation of the soluble fraction to carboxylates

The COD balance of the thin stillage fermentation revealed that membrane electrolysis resulted in a shift in the fermentation of VFA, from a majority C2 and C3 (64 ± 5 % C2 and C3, 36 ± 2 % C4–C6, as an average percentage of the total carboxylates on a COD basis during steady state, *n* = 10) to a majority of C4 to C6 (30 ± 5 % C2–C3, 70 ± 12 % C3–C6) without a change in the total amount of VFA on a COD basis (Fig. 3a). The proportion of acetate was lower in the applied current case than in the control, but only slightly and not significantly across all measured time points during the steady state (n = 10) (14 ± 2 % control, 8 ± 5 % with current). The proportion of propionate decreased from 51 ± 6 to 21 ± 7 % with current. The proportion of butyrate was greater under applied current (11 ± 2 % control, 25 ± 7 % with current), with a similar trend with valerate (25 ± 8 % control, 42 ± 11 % with current), and caproate (0 ± 1 % control, 3 ± 2 % with current). The extent of chain elongation can be compared as “chain elongation equivalents”, the concentration of carbon (gC L^−1^) at steady state that has been added through a chain elongation pathway on the theoretical assumption that all VFA start at either acetate (C2) or propionate (C3). The extent of VFA chain elongation at steady state in the applied current case was 2.6 ± 0.6 gC L^−1^ of chain elongation equivalents, significantly higher than the control case at 1.4 ± 0.4 gC L^−1^ (*t* test: *α* = 0.05, *p* = 1.9 × 10^−4^, *n* = 10).

Only a fraction of the feed was converted to VFA. This fraction is referred to here as the “reactive fraction” (Fig. 3b). The reactive fraction consisted of soluble cellulosic fragments (or ‘Sugars’), consisting of 4.4 ± 0.6 g L^−1^ glucose, 4.2 ± 0.5 g L^−1^ xylose and 2.9 ± 0.4 g L^−1^ arabinose (in total 12.3 ± 0.7 gCOD L^−1^); and glycerol (9.8 ± 2.4 gCOD L^−1^), lactate (2.1 ± 1.0 gCOD L^−1^), and C1 to C8 carboxylates (0.3 ± 0.2 gCOD L^−1^). The remainder of the thin stillage consisted of a solid fraction of proteins (8.9 ± 0.9 gCOD L^−1^) and other lignocellulosic solids (10.6 ± 2.5 gCOD L^−1^), a soluble COD fraction (sCOD) of protein (6.1 ± 1.3 gCOD L^−1^), and an assumed balance fats, oils and other biomass (4.2 gCOD L^−1^), in good agreement with a previous thin stillage characterization [27]. Membrane electrolysis did not alter any of the other main components characterized in this study (Fig. 3b). Zhou et al. studied glycerol fermentation with applied current, resulting in a mixed outcome with approximately 15–30 % of the carbon ending as VFA (mostly propionate), 20 % ending as biomass, 3–6 % as ethanol and 20–50 % as 1,3-propanediol [31]. Insignificant quantities of 1,3-propanediol were detected in this study, and propanol and butanol were detected at less than 1 gCOD L^−1^ each. Phenolic compounds were identified in the feed and fermentations at 1.2 g L^−1^. Both the control and applied current case showed no net increase of ethanol in the broth from 1.2 ± 0.6 gCOD L^−1^ fed to 1.7 ± 1.0 gCOD L^−1^ and 1.3 ± 0.1 gCOD L^−1^, respectively. Phenolic compounds were detected in the extractant in trace quantities.

Soluble cellulosic fragments, glycerol and lactate were consumed equally in both the control and experimental reactors (Fig. 3b). In the control, this resulted in predominantly acetate and propionate. Lactate is present in the fed thin stillage and can be used to elongate acetate to butyrate, as can ethanol [16]. Approximately 0.14 gC L^−1^ day^−1^ of lactate and 0.05 gC L^−1^ day^−1^ of ethanol enter the system by feeding. This fed lactate and ethanol can account for the chain elongation of C2 and C3 species to C4 and longer, assuming all carboxylates of C4 and longer are a result of chain elongation. The control case needs a total of 0.2 gC L^−1^ day^−1^ to elongate C2 and C3, compared with the applied current case which requires 0.7 gC L^−1^ day^−1^.

### Increased organic loading and current lead to increased VFA production

A brief, secondary experiment tested the system under doubled organic loading rate and a constant applied current of 100 mA, resulting in a 5.5 times increase in the VFA production rate to 10.4 ± 1.1 gCOD_VFA_ L^−1^ day^−1^. The disproportional increase in production can be partially attributed to removing the substrate limitation, in addition to the constant supply of hydrogen gas to the fermentation. The VFA production rate was 10.4 ± 1.9 g COD L^−1^ day^−1^ (3.3 ± 0.6 gC L^−1^ day^−1^), at an extraction of 26 ± 5 % of produced VFA. The conversion rate of sCOD was also higher at 60 ± 11 % of the total sCOD fed, or 86 ± 16 % of the reactive fraction, with a higher proportion of C6 and now also C7 VFA. In the previous experiment, heptanoic acid (C7) was not detected (Additional file 1: Figure SI 1). The constant current appears to have increased the total conversion of the reactive fraction. The greater supply of hydrogen gas (per liter of reactor volume), 66 mmol L^−1^ day^−1^ compared with 25 mmol L^−1^ day^−1^ in the first experiment, resulted in a lower concentration of butyrate at 8 ± 0 % and valerate at 19 ± 1 % of the C2–C7 VFA. The proportion of caproate was 11 ± 6 %, compared to 3 ± 6 % in the previous experiment, and heptanoate at 1 ± 0 % (Additional file 1: Figure SI 1). The chain elongation equivalents increased relative to hydrogen generation, though not proportionally (Additional file 1: Figure SI 2). This suggests that the high organic loading rate and high current case (Additional file 1: Exp II, Figure SI 2) were either under excess hydrogen or the hydrogen was escaping the system before it could be utilized. Short-term batch tests at a range of applied current (Additional file 1: Figure SI 2B) showed a similar trend for total carbon chain elongation equivalents generated in the broth, where even though the applied current is doubled from 100 to 200 mA, the total chain elongation equivalents only increased incrementally.

In this high loading, constant current experiment, the volume of the extractant was halved and operated in batch to demonstrate acid accumulation and mimic a more realistic recovery strategy. A maximum concentration of 11.7 gC L^−1^ was reached in the anolyte, compared with the maximum of 2.3 gC L^−1^ in the previous experiment (Additional file 1: Figure SI 1). Phenolic acids were concentrated at up to 2.5 g L^−1^. Phosphoric acid (H_3_PO_4_, pH <1) accumulated at up to a maximum of 4.1 g L^−1^ and hydrochloric acid to 1.6 g L^−1^.

### Membrane electrolysis favors hydrogen metabolizing fermenters

The fermentation was initiated without an inoculum, and thus only organisms native to the thin stillage were cultivated. *Lactobacillus* spp. represented a relative abundance between 96 and 99 % of the bacteria at time zero and in the thin stillage fed throughout the experiment, with *Hallella* sp. and others making up the balance (Additional file 1: Figure SI 5, Table SI 1). *Lactobacillus* spp. abundance swiftly diminished in the control case to a maximum relative abundance of 1 %, and moved to a dominance of *Hallella* sp., with a relative abundance of 65 % after 2 days and a maximum relative abundance of 94 %, and an average of 75 ± 21 % across the whole experiment. The *Hallella* sp. dominance was similar to the applied current case with an average abundance of 42 ± 26 % across the whole experiment. The next most abundant species in the control case were *Dialister* sp. and *Megasphaera* sp., both of the family Veillonellaceae. *Dialister* sp. had a relative abundance between 3 and 26 %, with an average across all measured time points of 13 ± 8 %. *Megasphaera* sp. had a relative abundance between 1 and 5 %, with an average of 3 ± 2 %. The applied current case contained *Dialister* sp. at a similar relative abundance to the control case at between 0 and 22 %, with an average of 11 ± 7 % across the whole experiment. The greatest difference between the control and experimental case arose from the abundance of *Lactobacillus* spp. and *Megasphaera* sp. The *Megasphaera* sp. was present with a relative abundance between 0 and 57 %, with an average of 15 ± 21 % (n = 9, at steady state). *Lactobacillus* spp. in the applied current case slowly decreased over the first 8 days and was then present between 6 and 17 % from day 8 to 24, in stark contrast to the control case of between 0 and 1 %. *Pectinatus sp.*, *Bifidobacterium* sp. and *Prevotella* sp. are also present in the applied current case at a relative abundance of up to at least 5 %, which is similar to the control case with the exception of *Pectinatus* sp. which never exceeded 0.7 %. The *Lactobacillus* spp. had a minimum abundance under applied current of 5.9 % and a maximum of 67.0 %, compared to the control case with a minimum of 0.0 % and a maximum of 1.3 % (ignoring *t* = 0, at which both had greater than 99 % abundance of *Lactobacillus* spp., identical to the feed). In the control case, the *Lactobacillus* spp. dropped from 99 % to a relative abundance of 1.3 % after 2 days. The time point of the greatest abundance of the *Megasphaera* sp. (57 %, Additional file 1: Figure SI 3 and SI 4) coincided with a slight increase in *Lactobacillus* spp. and an increase in chain elongation, albeit following a slump possibly related to competition with *Pectinatus* sp. (Additional file 1: Figure SI 3 and SI 4). Day 14 of the applied current fermentation coincided with a low point of chain elongation in the steady state fermentation, a high relative abundance of *Pectinatus* sp., and the *Hallella* sp. maximum. In a brief period where *Pectinatus* outcompeted *Megasphaera*, minimal C4–C6 carboxylates were produced and a peak of propionate was observed (Additional file 1: Figure SI 4).

Soluble cellulosic fragments, glycerol and lactate were consumed in both the control and experimental reactors (Fig. 3a). In the control, this resulted in predominantly acetate and propionate while with an applied current a greater concentration of mid-chain VFA was observed (Figs. 3a, 5). *Megasphaera* sp. can ferment glucose, lactate and short-chain VFA toward short to mid-chain VFA, alongside CO_2_ and H_2_ [18], while the majority of the other bacteria of high relative abundance produce short chain VFA and intermediates. *Hallella* sp, consistently the most abundant species, is known to produce acetate and succinate, that latter of which can be decarboxylated to propionate, or lactate [32]. *Dialister* is a non-fermenting bacillus [32] and its consistent abundance alongside *Hallella* suggests that it may have produced propionate from succinate. Neither *Hallella* nor *Dialister* have been associated with longer chain carboxylates [32, 33]. *Bifidobacterium* sp. produces lactate and acetate [34], *Prevotella* sp. mainly produces acetate and succinate, with a slight production of iso-butyrate and iso-valerate [35] while *Pectinatus* sp. is associated with the production of acetic and propionic acid [36, 37]. *Pectinatus* sp. has been observed to produce propionate from glycerol at a biological cathode [38].

Reverse β-oxidation VFA chain elongation with lactic acid has been described in *Megasphaera elsdenii* [16], and *Lactobacillus* spp., can produce lactic acid from a variety of substrates [39, 40]. The butyrate, valerate and caproate in both the control and applied current fermentation (Fig. 3a) were likely generated by *Megasphaera sp*. through the following pathway [18, 19]:

$$\begin{aligned} {\text{Lactate}}^{ - } + {\text{ Acetate}}^{ - } + \,{\text{H}}^{ + } \to n\text{-}{\text{Butyrate}}^{ - } + {\text{ CO}}_{2} + {\text{ H}}_{ 2} {\text{O}}; \, \varDelta {\text{G}}^{0} = - 59.43{\text{ kJ mol}}^{{{ - 1}\,\,}} {\text{at }}37\,^\circ {\text{C}} \hfill \\ {\text{Lactate}}^{ - } + {\text{ Propionate}}^{ - } + {\text{ H}}^{ + } \to {\text{Valerate}}^{ - } {\text{ + CO}}_{ 2} + {\text{ H}}_{ 2} {\text{O}} \hfill \\ {\text{Lactate}}^{ - } + {\text{ Butyrate}}^{ - } + {\text{ H}}^{ + } \to {\text{Caproate}}^{ - } + {\text{ CO}}_{2} + {\text{ H}}_{ 2} {\text{O}} \hfill \\ \end{aligned}$$

*Megasphaera* sp. stands out as one of few bacteria in these consortia with a high relative abundance that is known to generate mid-chain VFA, and there is evidence here to suggest that it was able to utilize hydrogen from membrane electrolysis to drive VFA chain elongation. *Megasphaera* sp. was the only species whose relative abundance correlated positively with the concentration of chain elongation equivalents (i.e. extent of VFA chain elongation) by the Pearson correlation test (*R* = 0.63, *p* = 0.04) in the applied current case, whereas no correlation can be seen in the control case (*R* = 0.05, *p* = 0.89). When compared in the RAST database, 100 % similarity was found with “Megasphaera NP3,” a sequenced species closely related to *Megasphaera elsdenii* (RAST, http://rast.nmpdr.org/). Megasphaera NP3 contains genes for fatty acid production, glycolysis/gluconeogenesis and β-oxidation metabolism (for VFA chain elongation), along with genes for four NiFe hydrogenase mettalocenter assembly proteins, which makes it a good candidate for the ability to metabolize hydrogen [41]. Organisms that are both capable of reverse β-oxidation VFA chain elongation and hydrogen oxidation could gain energy by an increase of intracellular hydrogen which leads to an increase in NADH and NADPH, thus driving VFA reduction [2, 16, 24, 25, 41, 42, 43]. In this case, the majority of hydrogen was generated extracellularly in situ by membrane electrolysis and then transported into the cell to drive the so-called electro-fermentation.

**Fig. 4:**
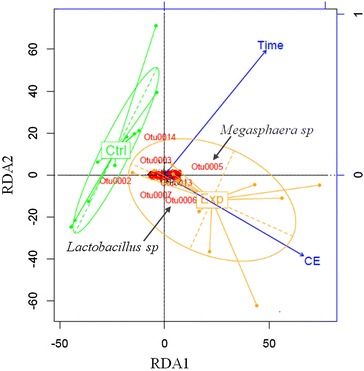
Redundancy analysis highlighting the dissimilarities among the relative abundances of the bacterial communities in the control and experimental (applied current) cases. “Ctrl” represents the community of the control reactor, which was described in RD2, while “Exp” indicates the community of the experimental (applied current) reactor, included in RD1. The *blue axis* represents time (days) and CE (extent of chain elongation)

A Redundancy Discriminant Analysis (RDA) was performed to examine the impact of applied current (experimental case) on the bacterial community, and their association with chain elongation over time, in comparison with a control (Fig. 4). We observed that the bacterial community in the applied current fermentation (Exp) was significantly different from that in the control case. The length of the chain elongation (CE) vector indicated a high relative association with the bacterial community in the Exp case (*r* = 0.561, RDA1 *p* value: 0.004). Within this community, Megasphaera sp. (Otu005) and Lactobacillus spp. (Otu006) are significantly correlated with chain elongation under the condition of the applied current (Exp), unlike in the control fermentation. In the control case, only time was significantly associated with the variations in the bacterial community (*r* = 0.778, RDA2 *p* value: 9.19 × 10^−5^). The position of each arrow (time or CE) with respect to the plot axis represents its degree of correlation with particular OTUs. In this way, the differences in the relative abundance of *Lactobacillus* spp. may be associated with CE, while the variations in *Megasphaera* sp. may as well tend to be influenced by time. The species-environment correlation values confirmed these observations (RDA1 = 0.649, RDA2 = 0.778).

**Fig. 5:**
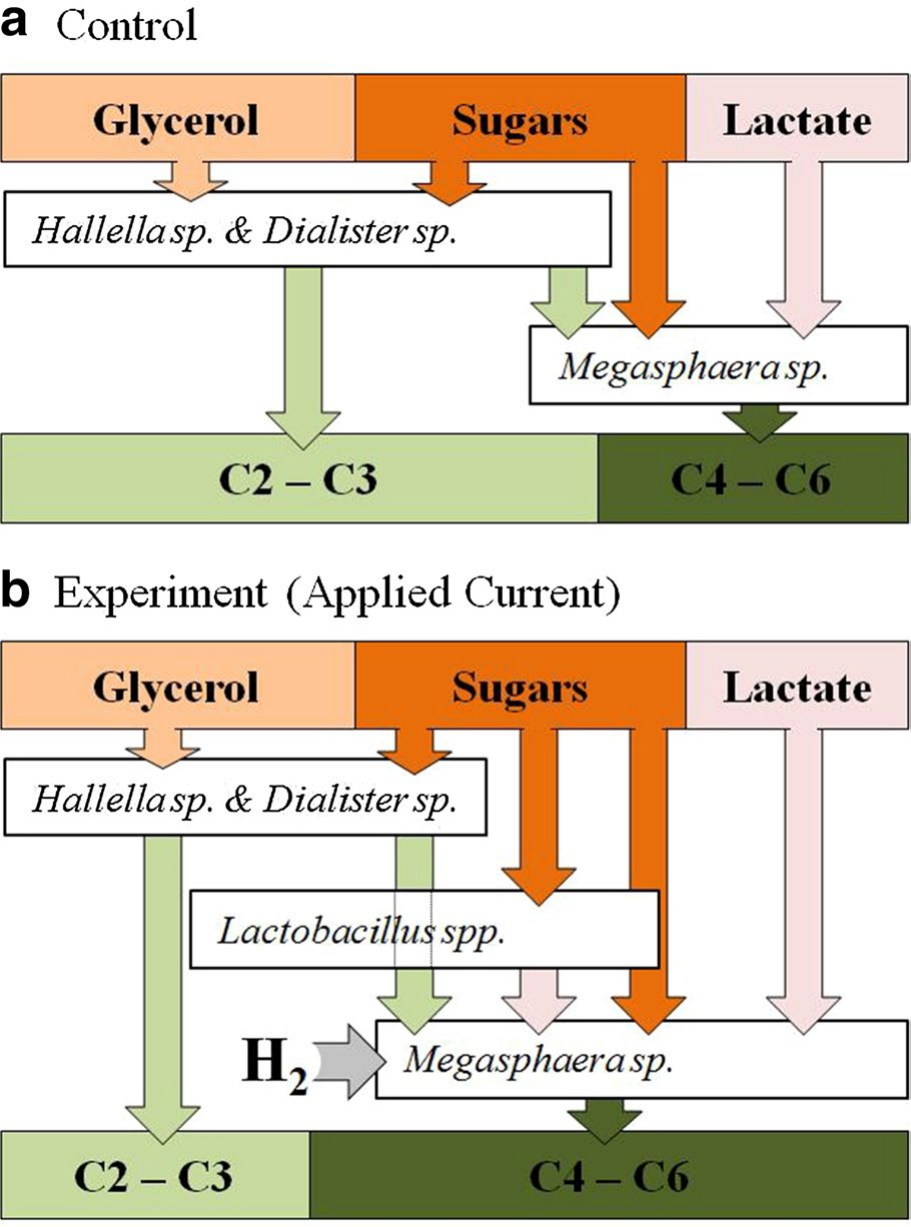
Schematic of species with the top four greatest relative abundance and the proposed pathway of substrates, VFA intermediate and VFA product. **a** In the control case some acetate (C2) and propionate (C3) may be used as an intermediate by *Megasphaera* sp. **b** In the experimental (applied current) case the *Megasphaera* sp. can metabolize electrolytically generated H_2_ to gain additional energy and through generating more reduced, longer chain VFA by lactate elongation

The relationship between *Lactobacillus* spp’s lactic acid production with the lactic, reverse β-oxidation and hydrogen utilizing capability of *Megasphaera* sp. enables the community to take advantage of the hydrogen generated in membrane electrolysis (Fig. 5). While *Lactobacillus* spp. are generally not recognized as hydrogen producers—or consumers—*Lactobacillus* spp. are often found in hydrogen producing consortia [44] and now here in a hydrogen consuming consortia. *Lactobacillus* spp. were always present in the applied current case during steady state at a relative abundance greater than 6 % at an average of 10.3 ± 3.4 % in the applied current case compared with an average relative abundance of 0.3 ± 0.4 % in the control (Additional file 1: Figure SI 3).

All species present in the fermentations were introduced from the feed (or whatever contamination followed). Phase contrast optical microscopy, flow cytometry and fluorescence in situ hybridization (FISH) confirmed that native species were present in the thin stillage despite the fact that thin stillage is retrieved between 75 and 80 °C, around the azeotrope of alcohol and water, downstream from the distillation column (Additional file 1: Figure SI 3). Enriching a native species from the target stream is attractive if the species fulfills the requirements of the process, though most chain elongation mixed culture studies use inocula with the intention of introducing *Clostridia*, more specifically *Clostridium kluyveri* [13, 16]. In our study *Clostridium* sp. are only observed in low abundance: less than 1 % in both the control and the experimental case. *Clostridium kluyveri* has been identified in other chain elongation studies, performing VFA chain elongation with an ethanol intermediate [12–17]. In one recent case, the *Clostridia* group IV was observed to generate caproic acid through a lactate substrate [45].

Understanding the principal actors in the community, be it in chain elongation or production of an intermediate, has implications in operational parameters such as temperature, residence time, pH and the substrate [46]. Selection of both substrate and strain are critical to ensure a targeted substrate conversion to the final product, be it in the conversion of oligosaccharides by *Lactobacillus spp*. [40], the production of caproic acid from sugars and lactic acid with *Megasphaera elsdenii* [18], or caproic production from acetic acid and ethanol with *Clostridium kluyveri* [12]. In the present state of technology, research generally focuses on product selectivity at the fermentation level, targeting optimum conditions toward the strain of interest [13, 15] with some examples of increased selectivity by developing extraction technology toward the product of choice, most notably with caproic acid due to its hydrophobicity [13, 17, 20].

### Co-products support biorefinery economics

The VFA as presented in this study are intended as an added value co-product, similar to distiller grains in bioethanol production, though their production can incur considerable base and acid costs. Membrane electrolysis is an immature technology and a complete economic analysis is premature, but the hydroxide output can be compared against the potential cost associated with caustic soda dosing in biological VFA production, particularly from organic waste streams. The control fermentation in this experiment required 0.83 kg caustic per 1 kg COD_VFA_ produced. Consider caproic acid as an attractive target product at around 1000 USD t^−1^ unrefined, and up to 2000–3000 USD t^−1^ refined (price assumptions here are based on discussions with industry partners and may vary). Assuming a conservative price of 300 USD t^−1^ of caustic, if the 0.83 kg caustic per 1 kg COD_VFA_ ratio holds and all the COD_VFA_ generated in this study could be directed to caproic acid (2.21 tCOD per 1 t caproic acid), then 1.83 t of caustic soda (550 USD) would be required for 1 t caproic acid. For unrefined caproic, this is more than half the market price, and 18.3–27.5 % for the refined caproic acid price range. pH control may indeed be further optimized, but this control fermentation on real thin stillage demonstrates the clear and present issue of a high caustic dosing requirement. Moreover, the caustic dosing is required to maintain the fermentation in the biocompatible range for production, and to acquire an acid product, considerable acidification may be needed. The production of OH^−^ and H^+^ by membrane electrolysis avoids caustic and acidic dosing, and also avoids salts entering the fermentation and extractant. The membrane electrolysis route has a cost associated with power input, which is approximately 2 kWh per kg COD_VFA_ based on these experiments. Assuming 0.05 USD kWh^−1^ electricity cost, this is around 100 USD t_COD_^−1^, though we stress this is specific to these experimental conditions. This includes the caustic correction and, unlike the control case, extraction and acidification. If the issue of solids buildup between the electrode and the membrane can be avoided and the theoretical power saving of 30 % holds, the power cost comes to 70 USD t_COD_^−1^. In principle this power input can come from renewables to improve the sustainability argument. Note that these calculations disregard downstream processing, which can be capital and energy intensive when separating compounds with similar properties, such as mixed VFA. For industrial, bulk chemicals, high product selectivity is critical to the economics of recovery and purification.

The reactive fraction of thin stillage is attractive as a target substrate as the cellulosic fragments and glycerol are readily convertible and have little value, though it is important not to detract from the contribution of these solids to existing production of DDGS. Avoiding degradation of solids is attractive in market in demand of livestock feed, as thin stillage is generally dewatered with the solids and syrup contributing to the production of DDGS, a low value but critical co-product whose value is linked to protein and fiber content. At this stage, it appears that membrane electrolysis allows most solids and proteins to pass without degradation. The apparent electro-coagulation effect may improve the prospects for solids recovery, though this is speculative, as the mechanism is scarcely documented.

## Conclusions

In this work, we have demonstrated the impact of membrane electrolysis, a chemical-free extraction technology, on the fermentation of an under-utilized, real biorefinery stream to generate reduced VFA. This study has shown that membrane electrolysis can result in an increased abundance of *Megasphaera* sp. and *Lactobacillus* spp. in thin stillage, resulting in an increase in VFA chain elongation through lactic acid, driven by in situ hydrogen generation. Membrane electrolysis can provide a driving force to select for hydrogen metabolisers such as *Megasphaera elsdenii* and potentially *Clostridiumkluyveri* or others. In addition to hydrogen, membrane electrolysis generates hydroxide ions which can replace the extensive caustic dosing typical of VFA production.

A central tenet of this work is to move from chemical and heat intensive petrochemical processes, as chemicals and heat both imply embedded petrochemical energy. Membrane electrolysis is electricity driven, and can only be a sustainable technology if this electricity can be sourced sustainably. To move forward, an electro-fermentation should be performed at industrially relevant production and recovery rates. The overall sustainability with respect to the input power and the materials of the system must be critically assessed to confirm that membrane electrolysis can give an added value to the fermentation and chain elongation of sustainable VFA. Beyond sustainability, practicality and economics of scale are crucial to a new industrial biotechnology. Minimizing the electrode and membrane material relative to fermenter volume, and maximizing efficient use of power input will be crucial to transition to an industrial scale, with some lessons to be learned from full scale electrochemistry such as electrodialysis and the Chlor-Alkali process.

## Methods

### Fermenter and electrochemical cell

In all experiments, semi-continuous fermentation was coupled with a continuous electrochemical membrane electrolysis. Multi-ported vessels of 1 L were used as fermenters (Glasgerätebau Ochs Laborfachhandel e.K., Germany) connected to electrochemical cells as described previously with a volume of 0.2 L per electrode chamber, internal dimensions 200 mm × 50 mm × 20 mm (depth) [23]. The total working liquid volume was 1.2 L, accounting for both the fermenter, the electrochemical cell and tubing. The anolyte was circulated from a 1 L schott bottle to the electrochemical cell, with an identical working volume of 1.2 L. The chambers were separated by an AEM (fumasep FAB, FumaTech GmbH, Germany). The cathode was an AISI Type 316L stainless steel wire mesh of 200 mm × 50 mm exposed working area with 564 µm mesh size, 140 µm wire thickness (Solana nv, Belgium), and the anode was an iridium mixed metal oxide coated titanium electrode (IrO_2_/TaO_2_: 0.65/0.35), 200 mm × 50 mm, with a centrally attached, perpendicular current collector (Magneto Special Anodes BV, The Netherlands). The catholyte and anolyte consisted in the fermentation broth and tap water (extractant), respectively. The electrolysis reactions consume water (catholyte, water reduction: H_2_O + e^−^ → ½ H_2_ + OH^−^; anolyte, water oxidation: H_2_O → 2e^−^ + 2 H^+^ + O_2_); however, assuming complete efficiency at the maximum current used here of 100 mA this would account for a 1.6 g of water per day from the catholyte and 0.8 g per day from the anolyte. Both were recirculated in their respective compartment at 6 L h^−1^ by peristaltic pump. In the control case, the pH was controlled between pH 5.4 (2 M NaOH dosing) and 5.7 (2 M H_2_SO_4_ dosing), whereas in the experimental case (with applied current), the pH was controlled between 5.4 and 5.7 by electrochemical water reduction and dosing with the acidified extractant in case of pH overshoot. The current was applied by a potentiostat (VSP, Biologic, France) in chronopotentiometry mode. The current was manually adjusted according to pH of the fermentation broth. Following feeding, an applied current of 100 mA (i.e. 10 A m^−2^) was applied for the first 20–24 h, or until pH 5.7 was reached. The pH controller would automatically dose acidic extractant from the previous period to return the pH below the set point. A maintenance period followed at which 20 mA was applied until the next feeding event as to minimize dosing of acidified extractant into the broth. All experiments were performed in a 35 °C temperature controlled room.

In the first experiment, 400 mL of the reactor volume was replaced with thin stillage at 2-day intervals for an equal HRT and solids retention time (SRT) of 6 days. The 6-day HRT/SRT was chosen to allow good conversion of substrate and sufficient time for adaption of the community to the conditions. The fermentation broth and the extractant were replenished at the same rate and the reactors were sampled every 2 days before feeding. After a steady state was reached the experimental cell was operated for four HRTs. The applied current was removed after 24 days to confirm the negation of the effect.

In the second experiment, the current was applied and the residence time of the fermentation broth was decreased to 3 days by replacing 400 mL of reactor volume daily, and sampling daily. The extractant volume was decreased to 600 mL from 1200 mL and operated in batch mode during this experiment, to mimic more realistic operation of extractant concentration and accumulation.

Supplementary batch tests explored a range of applied currents in which the reactor and extraction cell were filled with thin stillage and run for 6 days for each run. The reactors were emptied and refilled between each test. The applied current was set at 0.1 mA for the no current case, 50 mA (5 A m^−1^), 100 mA (10 A m^−1^) and 200 mA (200 A m^−1^) and the pH was controlled between pH 5.4 and 5.7 with 2 M NaOH and 2 M H_2_SO_4_. The AEM was replaced between each test and reactor components were scrubbed with a brush and tap water only to limit cross-contamination.

### Microbial characterization and community analysis

DNA extraction was performed using a PowerSoil DNA extraction kit (MO BIO Laboratories, USA) according to Roume et al. [47]. Biomass has first been concentrated by centrifugation in sterile 2 mL bead beating Micrewtubes (Simport, Canada) for 1 min at 20,238*g*. The protocol consists of cell lysis by bead beating in 2 mL Microtubes bead beating tubes and lysis buffer, in a Fast Prep-96 instrument (2 times 40 s at 1600 rpm) followed by removal by precipitation of diverse polymerase chain reaction (PCR) inhibitors according to the manufacturer’s instructions. Total DNA was captured on a silica membrane incorporated into a chromatographic spin column, washed and then eluted in the dedicated buffer. Concentration of double-stranded DNA was quantified using the QuantiFluor dsDNA system and measured with a GloMax 96 Microplate Luminometer (Promega GmbH, Germany). For quality control, the isolated DNA was amplified by PCR using Illumina sequencing primers and separated by electrophoresis.

The V3–V4 region of the bacterial 16S rRNA gene was sequenced with Illumina sequencing Miseq v3 Reagent kit (http://www.illumina.com/products/miseq-reagent-kit-v3.ilmn, by LGC Genomics GmbH, Berlin, Germany) using 2 × 300 bp paired-end reads and primers 341F-785R described by Stewardson et al. [48]. The PCRs included about 5 ng of DNA extract, 15 pmol of each forward primer 341F 5′-NNNNNNNNTCCTACGGGNGGCWGCAG and reverse primer 785R 5′-NNNNNNNNTGACTACHVGGGTATCTAAKCC in 20 µL volume of MyTaq buffer containing 1.5 units MyTaq DNA polymerase (Bioline) and 2 µL of BioStabII PCR Enhancer (Sigma). For each sample, the forward and reverse primers had the same 8-nt barcode sequence. PCRs were carried out for 30 cycles using the following parameters: 2 min 96 °C pre-denaturation; 96 °C for 15 s, 50 °C for 30 s, 72 °C for 60 s. DNA concentration of amplicons of interest was determined by gel electrophoresis. About 20 ng amplicon DNA of each sample was pooled for up to 48 samples carrying different barcodes. PCRs showing low yields were further amplified for 5 cycles. The amplicon pools were purified with one volume AMPure XP beads (Agencourt) to remove primer dimer and other small mispriming products, followed by an additional purification on MinElute columns (Qiagen). About 100 ng of each purified amplicon pool DNA was used to construct Illumina libraries using the Ovation Rapid DR Multiplex System 1-96 (NuGEN). Illumina libraries were pooled and size selected by preparative gel electrophoresis. Sequencing was done on an Illumina MiSeq using v3 Chemistry (Illumina).

Bioinformatics was executed with the mothur community analysis pipeline [49]. The analysis started from the primers clipped 16Sr RNA sequences; containing sequences where primers were detected and removed (allowing 2 mismatches) and turn into forward and reverse primer orientation which was later combined using the make.contigs command. The use of this open-source software package involved three sequential steps. The first step consists of the preparation and denoising of sequences, and extraction of the V3–V4 region. Low-quality sequences were removed and the frequency of sequencing and PCR errors reduced. The sequences were first trimmed using screen.seqs command (allowing no base name ambiguity and a maximum length of 427 bases). Sequences showing a weak alignment (allowing a maximum of four bases in homopolymer) with a V3–V4 customized SILVA database (v119) were removed as well as overhangs at both end of each sequence. The sequences were pre-clustered (allowing a maximum of 4 mismatches per sequence) and chimeric sequences were removed using UCHIME software [50] and sequences have been classifying using RDP v10 database (allowing at least a bootstrap value of 65 %) and sequences not identified as bacteria have been removed. The second step of the pipeline consisted of clustering sequences into operational taxonomic units (OTUs). OTU binning was completed using a hierarchical clustering algorithm implemented within mothur and considering a cutoff of 0.03. The third step involved assignment of the taxonomic information to sequences and OTUs. The analysis was carried out on randomly subsampled OTUs such that each file contained the same number of sequences (5401). The ade4, stat and psych packages of the R software (R version 2.13.2, http://www.r-project.org/) were used, respectively, for a principal component analysis (PCA; dudi.pca function), representation of the heatmap (heatmap function) and Pearson coefficient calculation (corr.test function).

For microbial activity analysis from the thin stillage, raw samples were evaluated under an optical microscope (Axioskop, Zeiss) by phase contrast and fluorescence. Fluorescence in situ hybridization (FISH) was performed as described by Anton et al. [52]. Samples were fixed with 4 % paraformaldehyde and stained with probes for general bacteria domain EUB 338 I, II and III with FLUO fluorophores.

### Stream characterization and analysis

In this study we refer to the linear, saturated monocarboxylate C2–C8 fatty acid conjugates collectively as VFA or individually by the common name of the dissociated anion, e.g. acetate, caproate, when discussing the compound in the more neutral fermentation broth. All carboxylate fermentation is from thin stillage of Alco Bio Fuel NV (Ghent, Belgium) (stored at 4 °C). No inoculum was added, the fermentation proceeded according to the bacterial community already present in the broth. New batches of stillage were periodically retrieved, with one batch requiring dilution to the appropriate COD range to maintain consistent organic loading. Stream characterization confirmed that after dilution the stream remained sufficiently consistent for these experiments, with some variation in solids content. Reported feed concentrations are averages of all feed streams including the diluted stream.

All samples were tested for TSS and VSS according to Standard Methods 2540D and E [51]. The C2–C8 fatty acids (including isoforms C4–C6) were measured according to Andersen et al. [22]. Management of pH was tracked by mass of acid or base dosed, and gas production was quantified with an external gas trap and assessed with a Compact Gas Chromatograph (Global Analyser Solutions, Breda, The Netherlands), equipped with a Molsieve 5A pre-column and Porabond column (CH_4_, O_2_, H_2_ and N_2_) and a Rt-Q-bond pre-column and column (CO_2_, N_2_O and H_2_S). Concentrations of gases were determined by means of a thermal conductivity detector. Eight samples were selected at random from the thin stillage feed samples for further characterization: four samples from the control effluent, four from the experimental effluent and four from the extractant during the steady state period with the average reported of the following analyses: total and soluble COD by Nanocolor^®^ kits (Macherey–Nagel GmbH, Germany); lactate, glycerol, 1,3-propanediol, ethanol, propanol and butanol by ion chromatography (Dionex DX 500); hemicellulosic and cellulosic fragments in the soluble phase (reported in this text as “soluble cellulosic fragments”, as to differentiate from (hemi)cellulosic material in the solids) by the NREL procedure according to Sluiter et al. [53], measured with high-performance liquid chromatography [Agilent Varian ProStar 220 SDM, USA; 5 mM H_2_SO_4_ mobile phase, 0.6 mL min^−1^ and 60 °C column temperature with a refractive index detector and Rezex H + column (Aminex)]; soluble and insoluble proteins by Kjeldahl nitrogen measurements according to Standard methods (4500-Norg B; APHA, 1992) [51], and calculated to protein COD content based on an assumed carbon to nitrogen ratio of 5:1. The protein measurement is intended only as an indication of changing protein concentrations and not intended as a strict protein quantification. The “Total Other Solids” is based on the difference between the measured total COD and the soluble COD, and its difference from the calculated insoluble protein COD. Non-organic anions chloride, nitrite, nitrate, sulfate and phosphate were determined on a 761 Compact Ion Chromatograph (Metrohm, Switzerland) equipped with a conductivity detector. The total phenolic content was assessed with the Folin Ciocalteu Assay method [54].

### Electrochemical analysis

The resistance of the whole cell was assessed by Current Interrupt [55]. 10 mA of current was applied at a period of 100 ms over 10 cycles successively, and the resulting voltage recorded at 0.2 ms intervals. The cell voltage change during the first interval of 0.2 ms is the ohmic drop of Current × Resistance, assuming that faradaic and diffusional processes present much slower relaxation times and therefore do not impact the voltage amplitude.

## Additional file

10.1186/s13068-015-0396-7Further information on composition and microbial community abundance in the applied current and control thin stillage fermentations.

## Abbreviations

VFA: volatile fatty acid
COD: chemical oxygen demand
sCOD: soluble chemical oxygen demand
DDGS: dried distillers grains with solubles
gC: grams of carbon, i.e. 1 mol of Caproic (C6) Acid = 116.16 g = 6 × 12.01 gC = 72.06 gC
HRT: hydraulic retention time
TSS: total suspended solids
PCR: polymerase chain reaction
