# Supplementary material for: Electrolytic extraction drives volatile fatty acid chain elongation through lactic acid and replaces chemical pH control in thin stillage fermentation
Source: Biotechnol Biofuels. 2015 Dec 21;8:221. doi: 10.1186/s13068-015-0396-7 (PMC4687354; doi:10.1186/s13068-015-0396-7)
Supplement: Supplementary file 1 — 10.1186/s13068-015-0396-7Further information on composition and microbial community abundance in the applied current and control thin stillage fermentations. [file 13068_2015_396_MOESM1_ESM.docx]

SUPPORTING INFORMATION

For

Electrolytic extraction drives volatile fatty acid chain elongation through lactic acid and replaces chemical pH control in thin stillage fermentation

Stephen J. Andersen, Pieter Candry, Thais Basadre, Way Cern Khor, Hugo Roume, Emma Hernandez-Sanabria, Marta Coma and Korneel Rabaey

Laboratory of Microbial Ecology and Technology (LabMET), Ghent University, Coupure Links 653, B-9000 Gent, Belgium

**Figure SI 1.** Comparison of the average outcome of VFA and production for the control case, experimental case [… ] and experimental case with increased loading rate and current. **Page 8**

**Figure SI 2.**Chain elongation equivalents vs. electrolytic hydrogen. **Page 9**

**Figure SI 3.** Cumulative line plot of species abundance over time. **Page 10**

**Figure SI 4.** Higher resolution perspective of *Megasphaera sp* and *Pectinatus sp* relative abundance and the short chain carboxylates concentration in the fermentations. **Page 11**

**Figure SI 5.** Redundancy discriminant analysis of the control and experiment, C1 to C6 VFA. **Page 12**

**Figure SI 5.** Micrographs from raw thin stillage. [… ] **Page 13**

**Table S1**. Measured and calculated chemical oxygen demand. All values are gCOD L^-1^ P**age 14**

**Table S2**. Other physico-chemical data (TSS, Kjeldahl Nitrogen, pH, Temperature, Conductivity) P**age 14**

**Table S3**. The top 11 most abundant bacterial species. P**age 15**


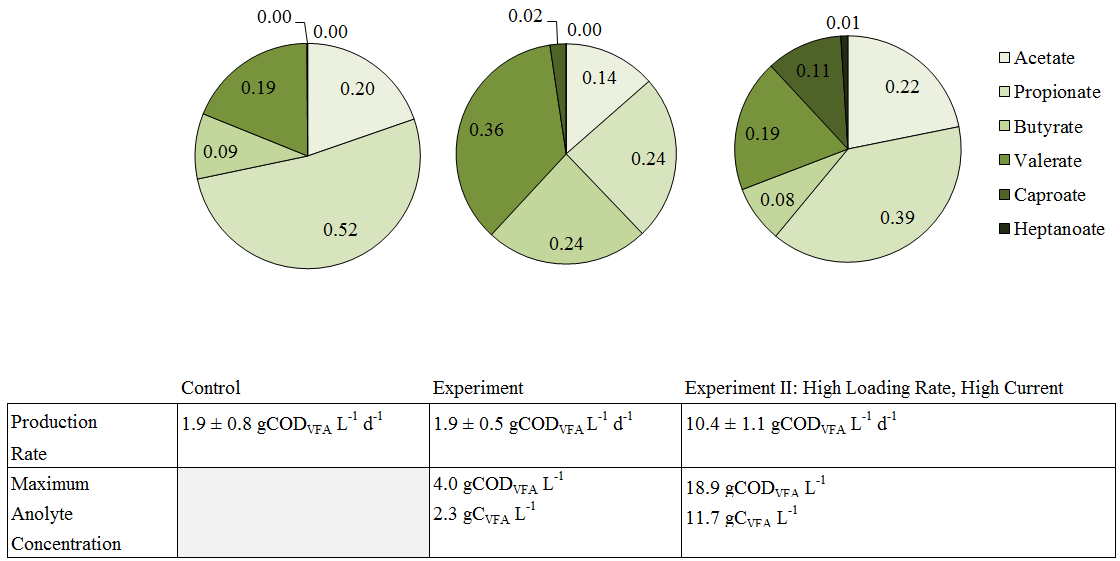


**Figure SI 1.** Comparison of the average outcome of VFA and production for the control case, experimental case (applied current) and experimental case with increased loading rate and current.


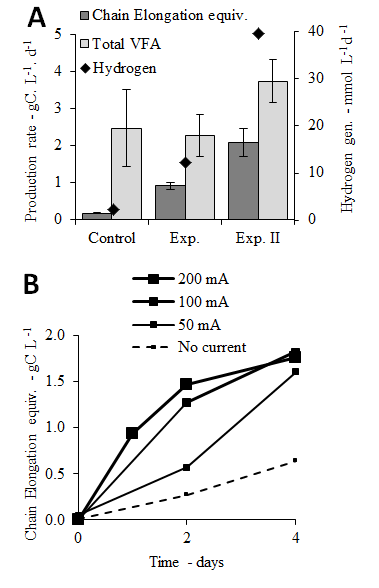


**Figure SI 2.** Chain elongation equivalents vs. electrolytic hydrogen. **A.** Production rate of total VFA compared against production of ethanol equivalents required for chain elongation to C4 to C7 carboxylates. Hydrogen gas reported is the average of that measured from the headspace and gas column. **B.** Chain elongation equivalents as measured in batch tests over time, for 0, 50, 100 and 200 mA.


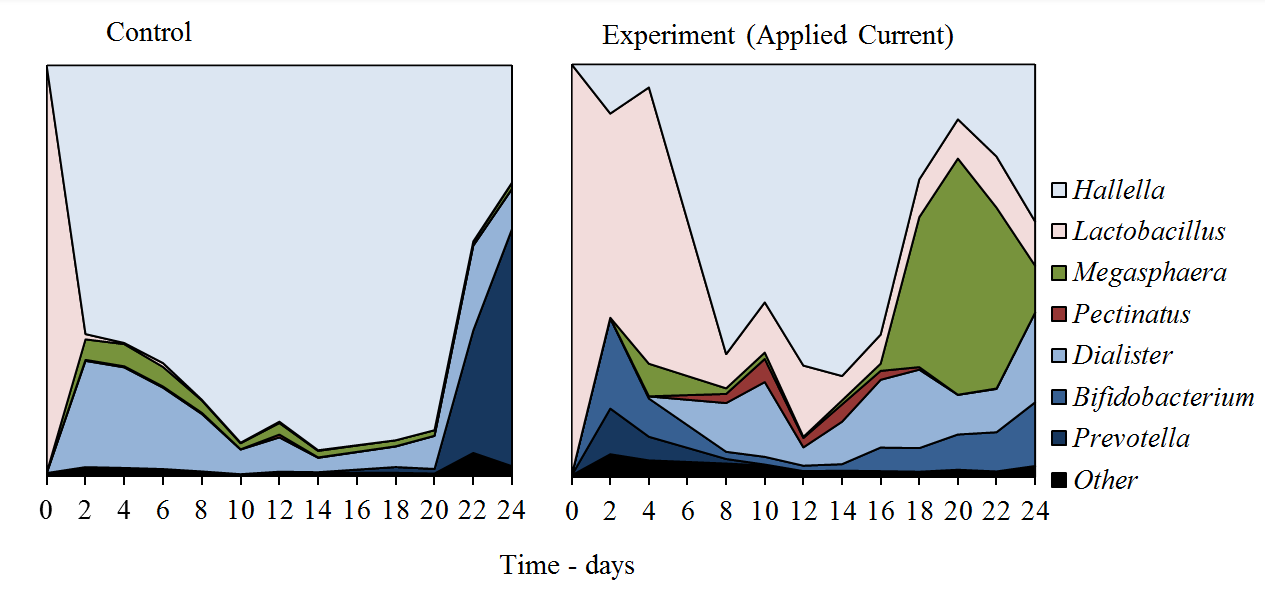


**Figure SI 3.** Cumulative line plot of species abundance over time. Data points at day 16 for the control and day 6 for the experiment are assumed values due to data collection error. Feed abundance is consistently in excess of 99% *Lactobacillus.*


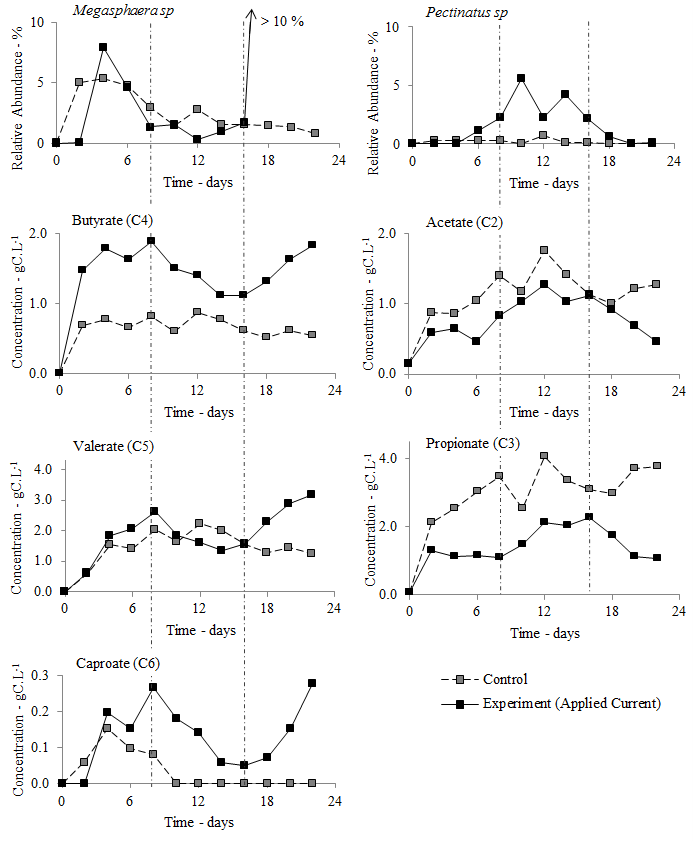


**Figure SI 4.** Higher resolution perspective of *Megasphaera sp* and *Pectinatus sp* relative abundance and the short chain carboxylates concentration in the fermentations.

**Figure SI 5.** Redundancy discriminant analysis of the control and experiment, C1 to C6 VFA.


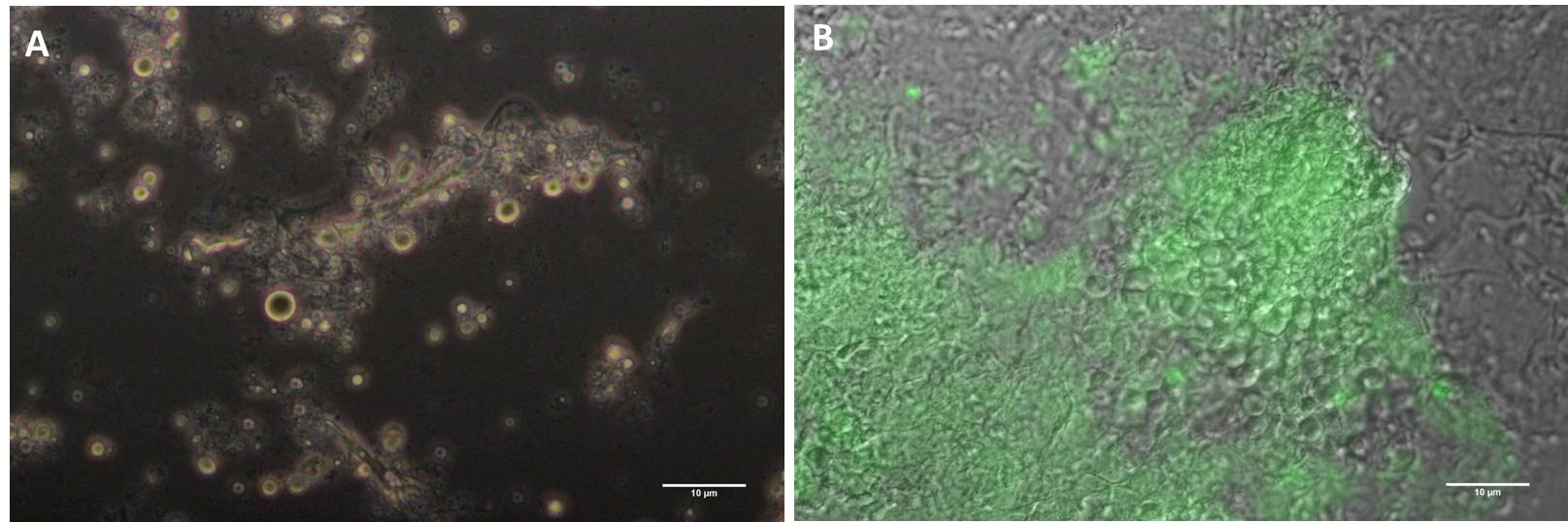


**Figure SI 6.** Micrographs from raw thin stillage. **A.** Contrast light image at 1000x. **B.** FISH image overlaid with transmitted light image. Green color stands for hybridization with EUB 338 I-II-II RNA probe. The scale bar signifies 10µm in both cases.

**Table SI 1.** Measured and calculated chemical oxygen demand. All values are gCOD L^-1^.

|  | **Feed** |  | **Control** |  | **Experiment** | | **Extracted** | |  |
| --- | --- | --- | --- | --- | --- | --- | --- | --- | --- |
|  | Average | StDev | Average | StDev | Average | StDev | Average | StDev | |
| **COD** | **55.50** | **10.60** | **52.30** | **4.50** | **43.30** | **10.10** | **9.32** | **0.30** | |
| sCOD | 36.00 | 10.10 | 32.80 | 4.50 | 22.33 | 3.10 | 9.32 | 0.30 | |
| **Total VFA** | **0.29** | **0.17** | **11.11** | **0.57** | **7.94** | **1.87** | **2.96** | **0.55** | |
| Acetate | 0.16 | 0.14 | 1.50 | 0.27 | 0.64 | 0.54 | 0.27 | 0.15 | |
| Butyrate | 0.01 | 0.03 | 1.22 | 0.20 | 2.00 | 0.50 | 0.75 | 0.21 | |
| Caproate | 0.00 | 0.00 | 0.02 | 0.07 | 0.27 | 0.17 | 0.06 | 0.07 | |
| Propionate | 0.08 | 0.26 | 5.63 | 0.64 | 1.67 | 0.72 | 0.65 | 0.10 | |
| Valerate | 0.04 | 0.16 | 2.74 | 0.90 | 3.36 | 1.35 | 1.23 | 0.40 | |
| **Reactive Fraction** | **25.38** |  | **2.19** |  | **1.94** |  | 0.00 |  | |
| Ethanol | 1.22 | 0.56 | 1.72 | 1.04 | 1.33 | 0.06 | 0.00 | 0.00 | |
| Sol. Cellu. Fragm. | 12.30 | 0.70 | 0.47 | 0.03 | 0.61 | 0.14 | 0.00 | 0.00 | |
| Glycerol | 9.74 | 2.43 | 0.00 | 0.00 | 0.00 | 0.00 | 0.00 | 0.00 | |
| Lactate | 2.12 | 0.97 | 0.00 | 0.00 | 0.00 | 0.00 | 0.00 | 0.00 | |
| **Solids** | **19.50** | **2.53** | **19.50** | **4.48** | **20.97** | **3.14** | **0.00** | **0.00** | |
| Insoluble Protein | 8.87 | 0.90 | 9.64 | 0.80 | 6.62 | 0.80 |  |  | |
| Total Other Solids | 10.63 |  | 9.86 |  | 14.35 |  |  |  | |
| **Other Solubles** | **10.62** |  | **19.51** |  | **13.45** |  | **6.36** |  | |
| Soluble Protein | 6.13 | 1.30 | 3.18 | 0.38 | 4.09 | 0.61 |  |  | |
| Other Solubles | 4.19 |  | 16.32 |  | 9.36 |  |  |  | |

**Table SI 2**. Other physico-chemical data. (TSS, Kjeldahl Nitrogen, pH, Temperature, Conductivity)

|  | **Feed** |  | **Control** |  | **Experiment** | |
| --- | --- | --- | --- | --- | --- | --- |
|  | Average | StDev | Average | StDev | Average | StDev |
| Total Suspended Solids, g L^-1^ | 20.33 | 2.84 | 18.62 | 1.86 | 11.17 | 1.70 |
| Total Kjeldahl Nitrogen, gN L^-1^ | 1.50 | 0.28 | 1.28 | 0.09 | 1.07 | 0.09 |
| Soluble Kjeldahl Nitrogen, gN L^-1^ | 0.64 | 0.04 | 0.32 | 0.04 | 0.41 | 0.07 |
| pH | 4.63 | 0.3 | 5.5 |  | 5.5 |  |
| Temperature, °C | 4 |  | 35 |  | 35 |  |
| Conductivity, mS/cm | 6.10 | 0.46 |  |  |  |  |
|  |  |  |  |  |  |  |

**Table S3**. The top 11 most abundant bacterial species

| Bacterial species (*sp.,* %) | Start of experiment | | | |
| --- | --- | --- | --- | --- |
|  | Control Feed | Control Eff | Exp Feed | Exp Eff |
| *Lactobacillus* | 99.09 | 0.17 | 99.65 | 8.29 |
| *Hallella* | 0.07 | 93.70 | 0.00 | 70.25 |
| *Dialister* | 0.00 | 3.50 | 0.00 | 11.81 |
| *Megasphaera* | 0.07 | 1.59 | 0.00 | 1.35 |
| *Pectinatus* | 0.02 | 0.09 | 0.00 | 2.22 |
| *Clostridium_sensu_stricto* | 0.06 | 0.17 | 0.00 | 0.17 |
| *Bifidobacterium* | 0.04 | 0.00 | 0.00 | 1.74 |
| *Prevotella* | 0.04 | 0.15 | 0.00 | 1.11 |
| *Propionibacterium* | 0.00 | 0.07 | 0.00 | 0.00 |
| *Desulfovibrio* | 0.00 | 0.00 | 0.00 | 2.28 |
| *Oscillibacter* | 0.00 | 0.00 | 0.00 | 0.07 |
| Other | 0.61 | 0.56 | 0.35 | 0.70 |

| Bacterial species (*sp.,* %) | End of experiment | | | |
| --- | --- | --- | --- | --- |
|  | Control Feed | Control Eff | Exp Feed | Exp Eff |
| *Lactobacillus* | 99.74 | 0.07 | 97.32 | 10.72 |
| *Hallella* | 0.06 | 28.51 | 2.15 | 38.10 |
| *Dialister* | 0.04 | 9.72 | 0.20 | 21.72 |
| *Megasphaera* | 0.00 | 1.43 | 0.02 | 11.33 |
| *Pectinatus* | 0.00 | 0.07 | 0.00 | 0.06 |
| *Clostridium_sensu_stricto* | 0.00 | 0.07 | 0.00 | 0.37 |
| *Bifidobacterium* | 0.00 | 0.00 | 0.00 | 15.44 |
| *Prevotella* | 0.00 | 57.79 | 0.00 | 0.13 |
| *Propionibacterium* | 0.00 | 0.02 | 0.09 | 0.00 |
| *Desulfovibrio* | 0.00 | 0.00 | 0.00 | 0.52 |
| *Oscillibacter* | 0.00 | 0.00 | 0.00 | 0.22 |
| Other | 0.17 | 2.31 | 0.22 | 1.39 |
